# Survival of Primary Human Hepatocytes and Death of Induced Pluripotent Stem Cells in Media Lacking Glucose and Arginine

**DOI:** 10.1371/journal.pone.0071897

**Published:** 2013-08-14

**Authors:** Minoru Tomizawa, Fuminobu Shinozaki, Takao Sugiyama, Shigenori Yamamoto, Makoto Sueishi, Takanobu Yoshida

**Affiliations:** 1 Department of Gastroenterology, National Hospital Organization, Shimoshizu Hospital, Yotsukaido City, Chiba, Japan; 2 Department of Radiology, National Hospital Organization, Shimoshizu Hospital, Yotsukaido City, Chiba, Japan; 3 Department of Rheumatology, National Hospital Organization, Shimoshizu Hospital, Yotsukaido City, Chiba, Japan; 4 Department of Pediatrics, National Hospital Organization, Shimoshizu Hospital, Yotsukaido City, Chiba, Japan; 5 Department of Internal Medicine, National Hospital Organization, Shimoshizu Hospital, Yotsukaido City, Chiba, Japan; University of Tampere, Finland

## Abstract

**Background:**

Tumorigenicity is an associated risk for transplantation of hepatocytes differentiated from human induced pluripotent stem (hiPS) cells. Hepatocytes express the enzymes galactokinase and ornithine transcarbamylase (OTC) to aid in their own survival. However, hiPS cells do not express these enzymes, and therefore, are not be expected to survive in a medium containing galactose and ornithine and lacking glucose and arginine.

**Materials and Methods:**

Real-time quantitative polymerase chain reaction (PCR) was performed to analyze the expression of galactokinase 1 (GALK1)1 and GALK2, ornithine carbamyltransferase, and phenylalanine hydroxylase (PAH). The hiPS cell line 201B7 was cultured in hepatocyte selection medium (HSM), which lacks glucose and arginine but contains galactose and ornithine. Furthermore, microscopic analysis of the cultured cells was performed after hematoxylin and eosin (H&E) staining, terminal deoxynucleotidyl transferase (TdT)-mediated dUTP nick end labeling (TUNEL). The hiPS cells were immunostained to assess their pluripotency in HSM. In addition, the primary human hepatocytes were cultured with or without hiPS cells in HSM.

**Results:**

The expression levels of *GALK1*, *GALK2*, *OTC*, and *PAH* in 201B7 were 22.2±5.0 (average ± standard deviation), 14.2% ±1.1%, 1.2% ±0.2%, and 8.4% ±0.7% respectively, compared with those in the adult liver. The hiPS cell population diminished when cultured in HSM and completely disappeared after 3 days. The cultured cells showed condensation or fragmentation of their nuclei, thereby suggesting apoptosis. TUNEL staining confirmed that the cells had undergone apoptosis. The 201B7 cells were positive for Nanog, SSEA-4, and TRA-1-60. The primary human hepatocytes survived when cultured alone in HSM and when co-cultured with hiPS cells.

**Conclusion:**

Therefore, HSM is and ideal medium for eliminating hiPS cells and purifying hepatocytes without inducing any damage.

## Introduction

Human induced pluripotent stem (iPS) cells have been established [1]. IPS cells are useful for drug discovery and regenerative medicine because they differentiate into somatic cells. If iPS cells could differentiate into hepatocytes, they would be useful for transplantation into patients with hepatic insufficiency. Ethical issues and graft-versus-host disease may be avoided with hiPS cells because they can be established in each patient individually. hiPS cells may therefore be an ideal cell source for patients. Hepatocytes are isolated from a fragment of resected donor liver with a 2-step collagenase perfusion [2]. Protocols are reported with regard to the differentiation of iPS cells to hepatocytes [3], [4]. The cells differentiated from iPS cells are hepatocye-like cells, not the same as primary human hepatocytes. It is necessary to use primary human hepaoctyes as a model of hepatocyes fully differentiated from iPS cells.

One of the problems of using iPS cell-derived cells for transplantation into patients is that they harbor the risk of tumorigenicity [5]. This tumorigenicity was initially attributed to genomic integration of viral vectors [6]. To reduce the risk, plasmid vectors have been used to introduce reprogramming factors such as Oct3/4, Sox2, and Klf4 [7]. The Sendai virus is used to establish iPS cells because there is no risk of altering of the host genome by the virus [8]. In addition the embryonic stem cell specific microRNA, miR-302, has been used to reduce the tumorigenicity of iPS cells by suppressing cyclin E-CDK2 and cyclinD-CDK4/6 [9]. Furthermore, Yakubov et al. introduced RNA synthesized from the cDNA of the four reprogramming transcription factors [10]. Combination of reprogramming factors have aslo been investigated. Nakagawa et al. omitted c-Myc to establish iPS cells, thereby reducing the tumorigenicity because c-Myc is a well-known oncogene [11]. Despite of the abovementioned efforts, the risk of tumorigenicity has not yet been eliminated. The link between pluripotency and tumorigenicity was reported in 1960 based on a study on teratocarcinoma [12]. The process of pluripotency and tumorigenicity involve self-renewal, proliferation, and active telomerase mechanisms [13]. Therefore, it is difficult to eliminate the risk of tumorigenicity if residual iPS cells persist in transplanted material. It is therefore necessary to develop methods to eradicate iPS cells surviving in differentiated somatic cell populations.

Glucose is an important source of energy for cell survival. Deprivation of glucose aids in the purification of hepatocytes because they produce this monosaccharide [14]. Pyruvate, which is the final product of glycolysis, enters the citric acid cycle. When pyruvate and glucose are removed from the medium, all neural cells die [15]. Galactose enters glycolysis as a substrate for galactokinase, which is expressed in the liver and kidney [16], [17]. Therefore, it is expected that hepatocytes can survive in a medium without glucose or pyruvate but containing galactose [18] [19].

Among all the amino acids, the removal of arginine is tolerated the least by cells cultured in vitro [20]. Arginine is produced through the urea cycle, which is exclusive to hepatocytes. Indeed, the removal of arginine led to the development of the first medium for purifying hepatocytes [14]. Tyrosine is produced by hepatocytes, and a subline of hepatoma cells has been established in a medium lacking serum, arginine and tyrosine [21]. The heptoma cell line has ornithine transcarbamylase (OTC) activity, which is involved in the urea cycle, and phenylalanine hydroxylase (PAH) activity, which produces tyrosine and is found in the liver and kidney [22]. Consequently, hepatocytes could be purified from embryonic stem (ES) cells in a medium lacking arginine and tyrosine.

We have previously developed a hepatocyte selection medium (HSM), which lacks glucose and arginine but contains galactose and ornithine [23]. The HSM was supplemented with dialyzed fetal calf serum (FCS) to ensure the complete absence of glucose and arginine. The medium enriched hepatoblast-like cells that showed up to 88% indocyanine green uptake from differentiating mouse embryonic stem (ES) cell cultures.

We anticipated that iPS cells would not survive and primary human hepatoyctes would survivie in the HSM. We, therefore, tested whether the HSM could eliminate these cells. We also addressed the question that the HSM could separate primary human hepatocytes cocultured with iPS cells. Primary human hepatocytes were used as a model of hepatocytes fully differentiated from iPS cells because we did not have a protocol of producing hepatocytes from iPS cells.

## Materials and Methods

### Cell Culture and Cell Counts

The hiPS cell line 201B7 (RIKEN Cell Bank, Tsukuba, Japan) was cultured feeder-free in ReproFF medium (Reprocell, Yokohama, Japan) in dishes (Asahi Techno Glass, Funabashi, Japan) with a thin coating of matrigel (Becton Dickinson, Franklin Lakes, NJ). These cells were kept in 5% CO_2_ at 37°C in a humidified chamber, and harvested with Accutase (Innovative Cell Technologies, Inc., San Diego, CA) for experiments. The thin coating was obtained by spreading a mix of 0.3 ml of matrigel with 8.7 ml of DMEM-F12 medium onto dishes, and incubating the dishes at room temperature for three hours. The morphological features of the cells were observed under a microscope (CKX41N-31PHP; Olympus, Tokyo, Japan). The cells were photographed in 5 different fields, and the number of cells each well were counted. The average of cell number was determined using five different fields each well. The average for three wells was used as the cell number (n = 3). The cells were cultured on 4-well culture slides (Becton Dickinson) coated with matrigel (thin coating) for hematoxylin and eosin (H&E) staining and terminal deoxynucleotidyl transferase (TdT)-mediated dUTP nick end labeling (TUNEL) and observed under an AX80 microscope (Olympus).

### Human Primary Hepatocyte Culture

Primary human hepatocytes were purchased from Lonza (Walkersville, MD) and cultured following the manufacturer’s instructions. Briefly, hepatocytes were thawed and spread onto CellBIND 24-well plates coated with type 1 collagen from the bovine dermis (Koken Co., Ltd., Tokyo, Japan) with hepatocyte culture medium (HCM) (Lonza) at a density of 150,000 cells/cm^2^. The HCM was prepared with 500 ml of hepatocyte basal medium (Lonza) supplemented with ascorbic acid, bovine serum albumin, fatty acid free, hydrocortisone, human epidermal growth factor, transferring, insulin, gentamicin/amphotericin-B (SingleQuatKit) (Lonza). The type 1 collagen coating was obtained by dissolving 0.3 ml of type 1 collagen in a hydrochloride solution at pH 4.0 at a density of 0.3 mg/ml, spreading the solution into each well of the 24-well plates, and incubating at room temperature. 30 min later, the type 1 collagen solution was removed and the wells were washed twice with phosphate buffered saline (PBS). The manufacturer’s instruction described that routine characterization of the hepatocytes includes positive albumin synthesis, 7-Ethoxyresorufin O-deethylation (EROD) and 7-ethoxycoumarin O-deethylation (ECOD) as described in the instruction.

### Co-culture of Primary Hepatocyte Culture and 201B7

201B7 cells and human primary hepatocytes were co-culutred as follows. Human primary hepatoctyes were spread onto 24 well plates and cultured in HCM for 24 hours as described above. The 201B7 cells were spread in the wells at a density of 3×10^4^/well. After 24 hours of culture with ReproFF, the medium was changed to HSM. The cells were observed under a CKX41N-31PHP (Olympus).

### Hepatocyte Selection Medium

The HSM was prepared from amino acid powders following the formulation of Leibovits-15 medium (Life Technologies, Grand Island, NY) with the omission of arginine, tyrosine, glucose, and sodium pyruvate but with the addition of galactose (900 mg/l) (Wako), ornithine (1 mM) (Wako), glycerol (5 mM) (Wako), and proline (260 mM) (Wako). Proline (30 mg/l) was added because it is necessary for DNA synthesis [24]. Aspartic acid was not included since it is one of the products of ornithine and a substrate of arginine. Knockout serum replacement (KSR) (Life Technologies) was added at a final concentration of 10% and was used instead of FCS to establish defined xeno free conditions. Depending on the experiment, KSR was dialyzed against phosphate buffered saline (PBS) to remove the amino acids and glucose.

### Real-time Quantitative Polymerase Chain Reaction

Total RNA (5 µg), which was isolated using Isogen (Nippon Gene, Tokyo, Japan), was used for the first-strand cDNA synthesis with SuperScript III and oligodT following the manufacturer’s instructions (Life Technologies). RNA from normal human fetal and adult liver was purchased from Clontech. Real-time quantitative PCR was performed with Fast SYBR Green Master Mix (Life Technologies), and the results were analyzed using the Mini Opticon system (Bio-Rad, Hercules, CA). Real-time quantitative PCR was performed for 40 cycles, using 5 s for denaturation and 5 s for annealing–extension. The primer pairs for real-time quantitative PCR of ribosomal protein L19 (RPL19), GALK1, GALK2, OTC, and PAH were 5′-CGAATGCCAGAGAAGGTCAC and 5′-CCATGAGAATCCGCTTGTTT (GeneBank: BC000530, expected product size: 157 bp), 5′-TGCTGTGCCTGGGGTTTATG and 5′-GCTGCTTGAGAGAGGTAGAAGGTG (NM_000154, 153 bp), 5′-TCACGACTTACTGGAGCAGGATG and 5′-CAAAACCAAAGCCCCACCTC (NM_002044, 177 bp), 5′-GGACATTTTTACACTGCTTGCCC and 5′-TCCACTTTCTGTTTTCTGCCTCTG (BC107153, 105 bp), and 5′-TGTCCATGAGCTTTCACGAG and 5′-TTAAAACCAGGGTGGTCAGC (NM_000277, 135 bp), respectively.

### TUNEL Staining

Apoptotic cells were detected using the Apoptosis in situ Detection Kit Wako (Wako Pure Chemicals, Osaka, Japan). The analysis of apoptotic cells was based on the TUNEL procedure, which consists of the addition by TdT to the 3′ termini of apoptotically fragmented DNA followed by an immunochemical detection using an anti-fluorescein antibody conjugated with horseradish peroxidase and diaminobenzidine (DAB) as a substrate.

### Immunostaining

Cells cultured on 4-well chamber slides (Becton Dickinson) coated with matrigel were fixed in 4% paraformaldehyde (Sigma Aldrich, St. Louis, MO) and incubated with hydrogen oxide in 100% methanol for 30 min at 4°C. Specimens were incubated with 2% fetal bovine serum in PBS (wash buffer) for 30 minutes at 4°C. Anti-Nanog (Repro Cell) antibody specimens were incubated in 0.1% sodium citrate (Wako Pure Chemicals) and 0.1% Triton X-100 (Wako Pure Chemicals) in distilled water. Cells in 1∶500 diluted anti-Nanog, anti-SSEA-4 (Millipore, Billerica, MA), and anti-TRA-1-60 (Millipore) were incubated in the wash buffer overnight at 4°C. After washing thrice with PBS, 500× diluted horseradish peroxidase labeled anti-mouse (GE Healthcare, Pittsburg, PA) or anti-rabbit antibodies (GE Healthcare) were incubated in the wash buffer for 3 hours at 4°C. Diaminobenzidine (DAKO, Glostrup, Denmark) was applied, and the nuclei were stained with hematoxylin (Muto Pure Chemicals, Tokyo, Japan) for 15 seconds. Specimens were observed and photographed with AX80 (Olympus, Tokyo, Japan).

## Results

**Figure 1:**
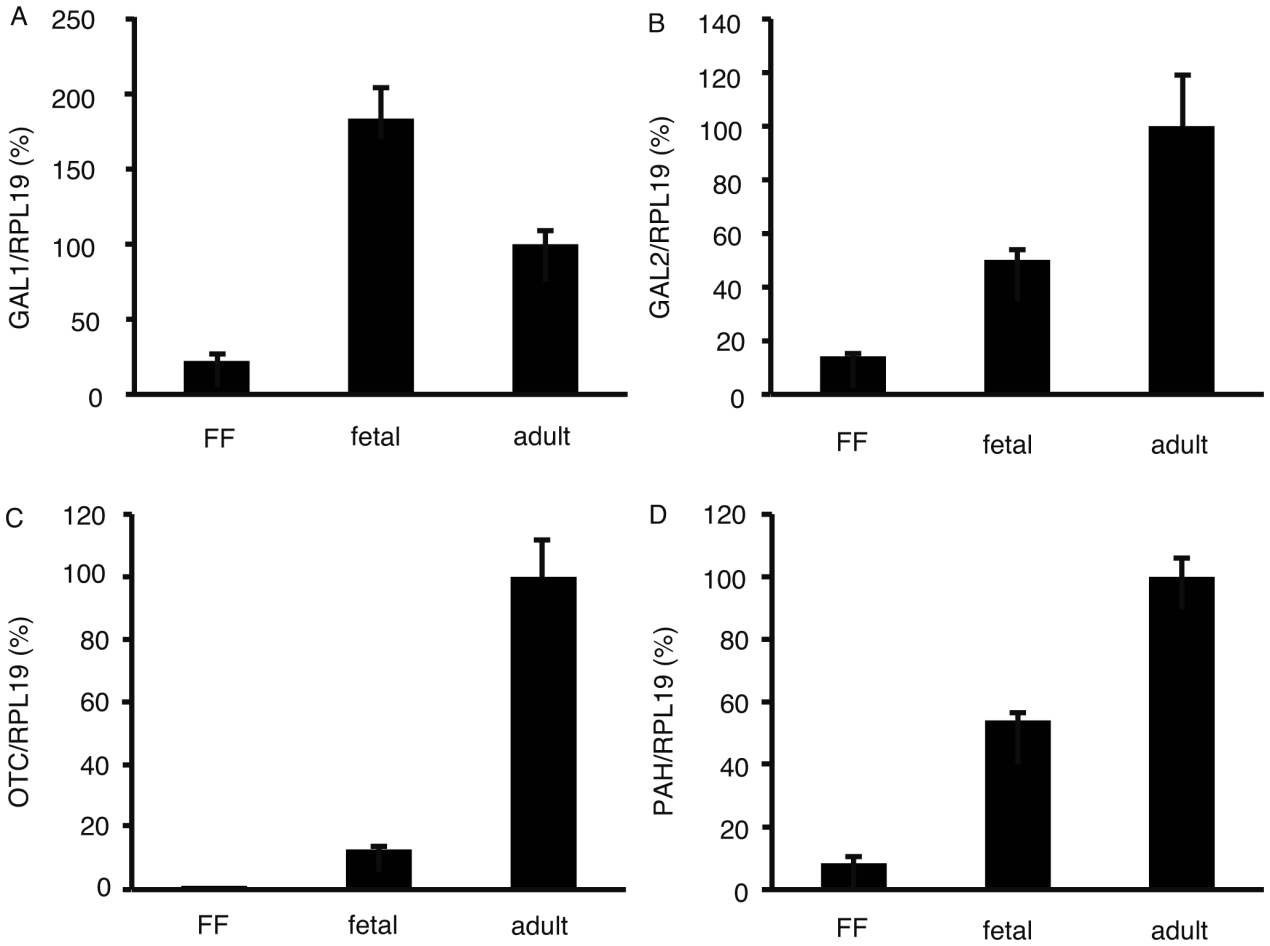
Real-time quantitative polymerase chain reaction (PCR). Expression levels of GALK1 (A), GALK2 (B), OTC (C), and PAH (D) were analyzed using real-time quantitativePCR. The expression levels were normalized against ribosomal protein L19. The relative expression levels of the 201B7 cells cultured with ReproFF were assigned the value 1. RPL19: ribosomal protein L19; GALK1: galactokinase 1; GALK2: galactokinase 2; OTC: ornithine transcarbamylase; PAH: phenylalanine hydroxylase; FF: 201B7 cells cultured with ReproFF, fetal: fetal liver, adult: adult liver; error bar: standard deviation, n = 3.

The expression levels of GALK1, GALK2, OTC, and PAH in the 201B7 cell line, fetal liver, and adult liver were compared (Figure 1). The expression levels of GALK1, GALK2, OTC, and PAH in the 201B7 cell line were 22.2% ±5.0% (average ± standard deviation), 14.2% ±1.1%, 1.2% ±0.2%, and 8.4% ±0.7%, respectively, compared with those in adult liver. Further, the expression level of OTC was significantly lower in the 201B7 cells than in the fetal and adult livers, which prompted us to culture 201B7 cells in HSM to investigate their survival rates.

**Figure 2:**
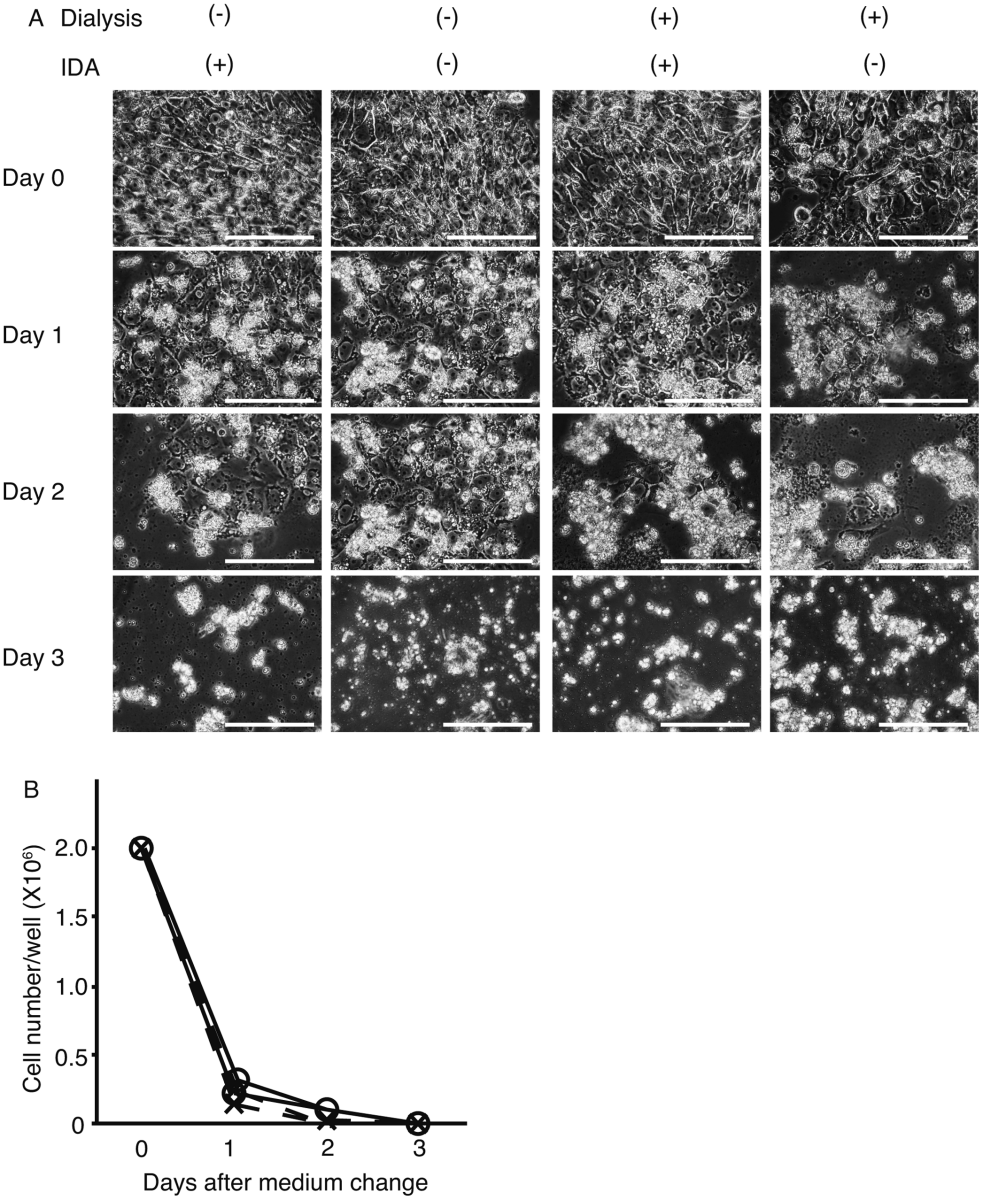
Morphological changes in 201B7 cells in hepatocyte selection medium (HSM). The medium was changed to HSM with or without knockout serum replacement dialysis and insulin, dexamethasone, and aprotinin (IDA) solution (A). Three days after changing the media, the cell numbers decreased to 0 (B). Open circle with solid line: dialysis (−) IDA (+); cross with solid line: dialysis (−) IDA (−); open circle with dashed line: dialysis (+) IDA (+); and cross with dashed line: dialysis (+) IDA (−). Original magnification: ×400; scale bar: 25 µm, n = 3.

The 201B7 cells were cultured on 6-well plates coated with matrigel in ReproFF. When they reached 90% confluency, the medium was changed to HSM, which contained either dialyzed (+) or nondialyzed (−) KSR was supplemented with or without insulin, dexamethasone, and aprotinin (IDA) solution. After changing the medium to HSM, the morphological features of the cells were analyzed (Figure 2 A). The 201B7 population size decreased in all types of HSM, and after 3 days, the cells had completely disappeared. The cell numbers significantly decreased toward day 3 after changing the medium (Figure 2 B).

**Figure 3:**
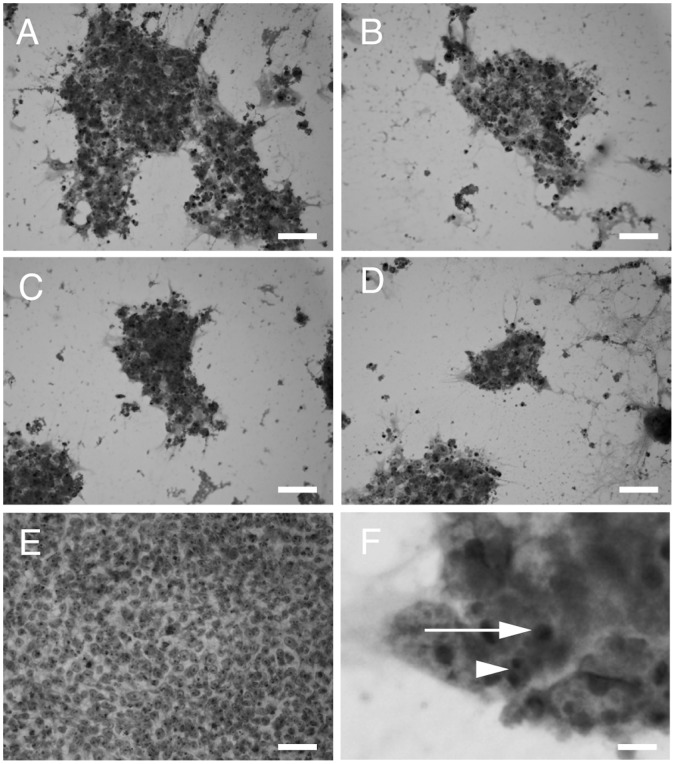
Hematoxylin and eosin staining. 201B7 cells were stained with hematoxylin and eosin at 1 day after the media change. The cells appeared damaged in the hepatocyte selection medium with or without dialysis of knockout serum replacement or use of insulin, dexamethasone, and aprotinin (IDA) solution (A, B, C, D). The cells appeared to be in comparatively good condition in ReproFF (E). The damaged cells showed condensation (F, arrow) or fragmentation of nuclei (F, arrowhead). A: dialysis (−) IDA (+); B: dialysis (−) IDA (−); C: dialysis (+) IDA (+); D: dialysis (+) IDA (−); E: ReproFF; and F: magnified view of D. Original magnification: ×200; scale bar: 25 µm (A, B, C, D, E) and 2.5 µm (F).

H&E staining was performed to analyze the morphological features of the 201B7 cells at 1 day after changing the medium to assess the effects of the different HSM conditions on the cells (Figure 3). Day 1 was chosen for analysis because the 201B7 colonies were too small to analyze after 2 or 3 days. The nuclei of the damaged cells were condensed or fragmented, which is characteristics of apoptosis. Notably, the 201B7 cells were viable in ReproFF. These results suggested that the 201B7 cells underwent apoptosis in the HSM.

**Figure 4:**
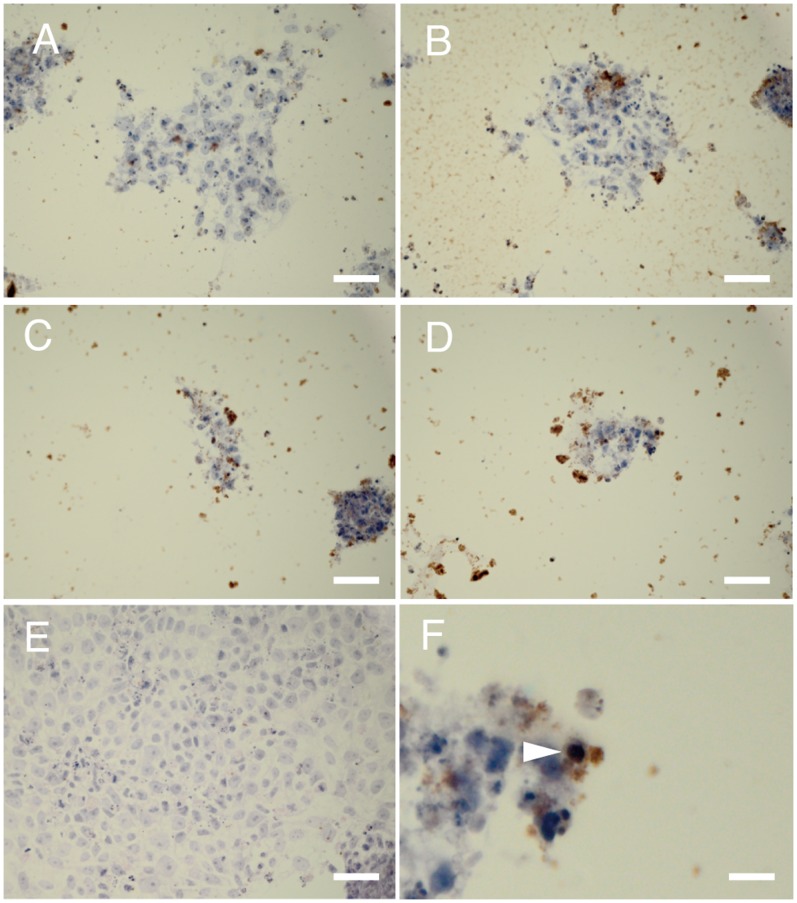
Terminal deoxynucleotidyl transferase (TdT)-mediated dUTP nick end labeling (TUNEL) staining. 201B7 cells were subjected to TUNEL at 1 day after the medium was changed to hepatocyte selection medium (HSM) with or without dialysis of knockout serum replacement or use of insulin, dexamethasone, and aprotinin (IDA) solution. Positive cells were seen in the different HSM conditions (A, B, C, D), whereas no cells were positive in ReproFF (E). The nuclei of the positive cells were condensed (F). A: dialysis (−) IDA (+); B: dialysis (−) IDA (−); C: dialysis (+) IDA (+); D: dialysis (+) IDA (−); E: ReproFF; and F: magnified view of D. Original magnification: ×200, scale bar: 25 µm (A, B, C, D, E) and 2.5 µm (F).

TUNEL staining was performed to confirm that the 201B7 population numbers decreased due to apoptosis (Figure 4). The cells cultured under different HSM conditions showed positive for TUNEL staining, whereas the cells in ReproFF showed negative results for TUNEL staining.

**Figure 5:**
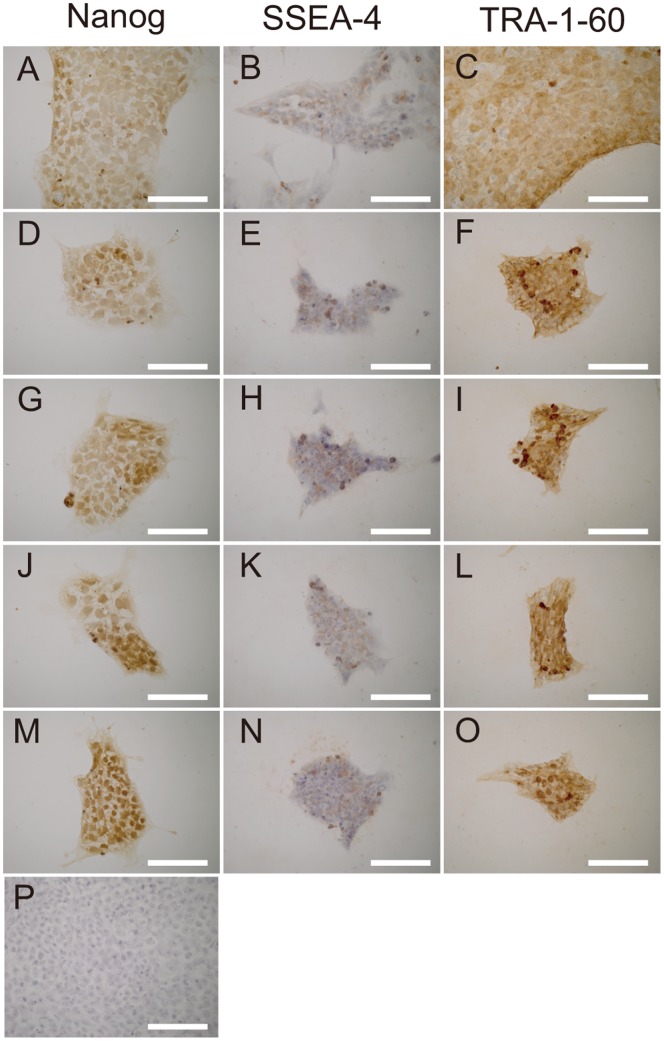
Immunostaining. 201B7 cells were subjected to immunohistochemistry at 1 day after the medium was changed to hepatocyte selection medium (HSM), with or without dialysis of knockout serum replacement or use of insulin, dexamethasone, and aprotinin (IDA) solution. All the cells were positive. A, B, C: ReproFF, D, E, F: dialysis (−), insulin (+); G, H, I: dialysis (−), insulin (−); J, K, L: dialysis (+), insulin (+); D: dialysis (+), insulin (−). P: negative control. Original magnification: ×200; scale bar: 25 µm.

To assess pluripotentcy, the 201B7 cells were stained with Nanog, SSEA-4, and TRA-1-60. At 24 hours after plating, the medium was changed to HSM, which contained either dialyzed (+) or nondialyzed (−) KSR was supplemented with or without insulin, dexamethasone, and aprotinin (IDA) solution. Positive results were obtained for Nanog in the nuclei of cells cultured with ReproFF and all types of HSM (Figure 5A, D, G, J, and M). Positive results were obtained for SSEA-4 (Figure 5B, E, H, K, and N) and TRA-1-60 (Figure 5C, F, I, L, and O) in the cytoplasms of cells cultured with ReproFF and all types of HSM.

**Figure 6:**
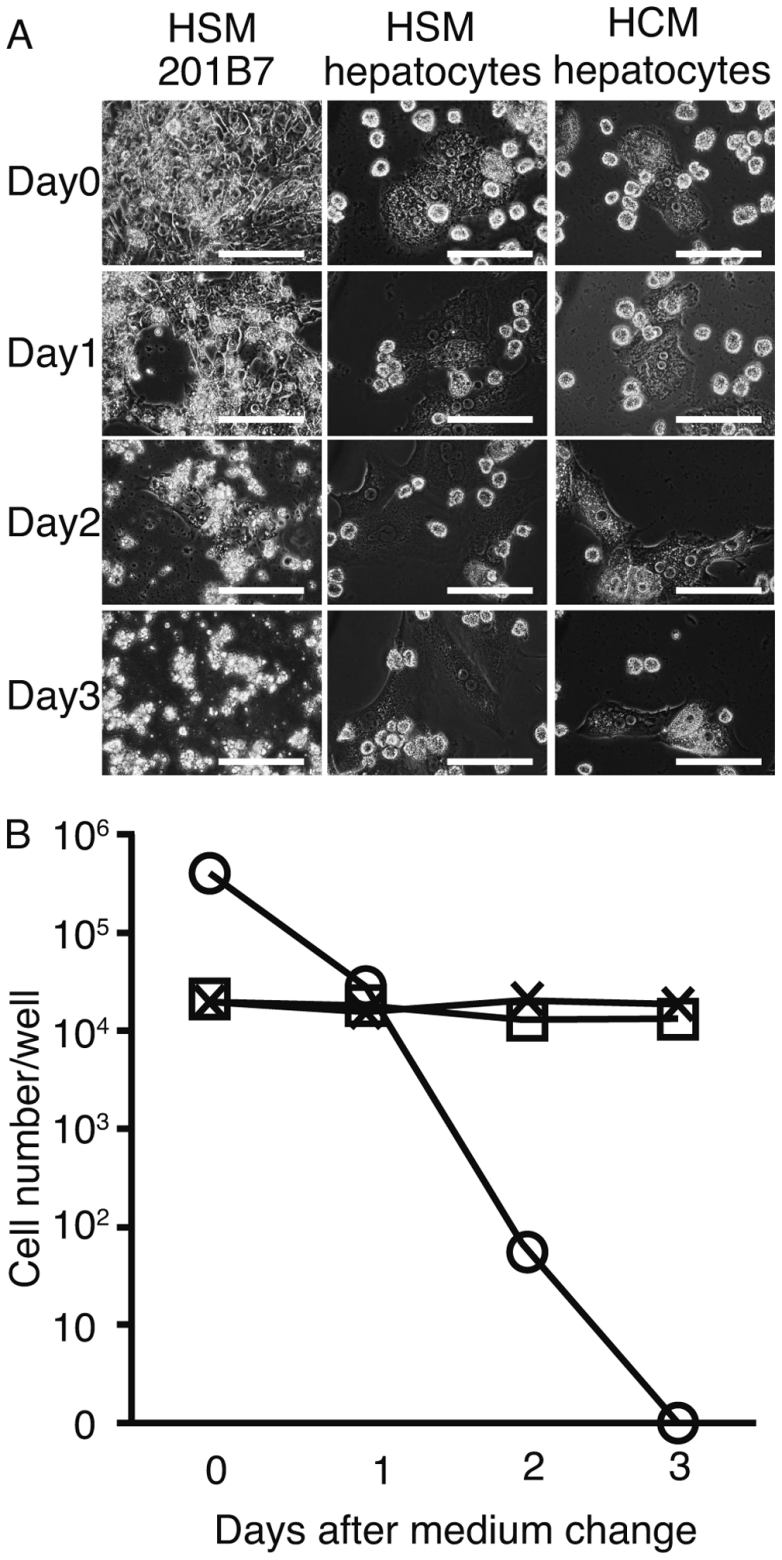
Primary human hepatocyte culture using hepatocyte selection medium (HSM). 201B7 cells or primary human hepatocytes were cultured in hepatocyte selection medium (HSM) with dialysis of knockout serum replacement dialysis and without insulin, dexamethasone, or aprotinin solution (A). The 201B7 cell count decreased (B). In comparison, the counts of the primary hepatocytes did not differ between the HSM and hepatocyte culture medium (HCM) (recommended medium for culturing primary hepatocytes. Note that the vertical Y-axis is logarithmic in B. Original magnification: ×400; scale bar: 25 µm. B: circle: 201B7, cross: hepatocytes in HCM, rectangular: hepatocytes in HSM, n = 3.

Human primary hepatocytes were cultured in HSM to compare their survival with that of the 201B7 cell line. The culture conditions were changed to the 4 different HSM conditions (Figure 6 A). The 201B7 cells started to disappear after 1day (Figure 6 B) whereas the primary hepatocyte cell count in HSM remained at the same level as in the HCM (Figure 6 B).

**Figure 7:**
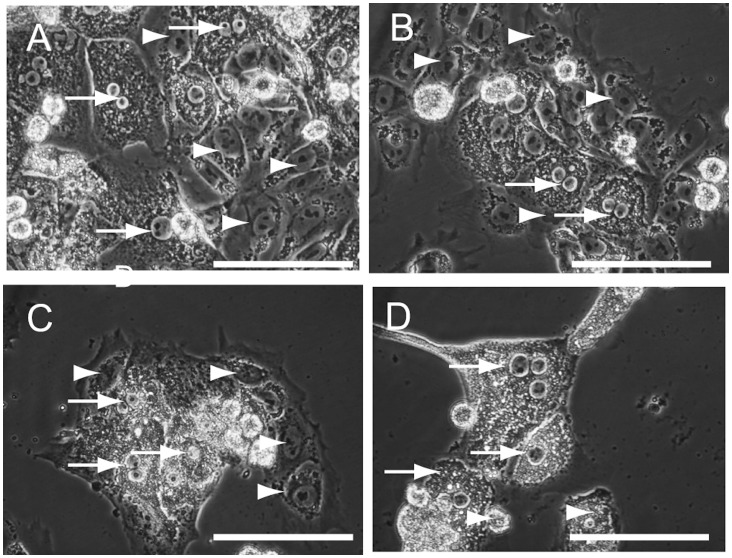
Co-culture of human primary hepatocytes and 201B7 in hepatocyte selection medium (HSM). After establishment of a co-culture of 201B7 and human primary hepatocytes (A), the medium was changed to HSM with dialyzed knockout serum replacement dialysis and without insulin, dexamethasone, or aprotinin. The 201B7 cells (arroheads) disappeared while the primary hepatocyes (arrows) were still alive at day 1 (B), day 2 (C) and day 3 (D). Original magnification: ×400, scale bar: 25 µm, arrow: 201B7 cells, arrowhead: hepatocytes.

Finally, human primary hepatocytes were cocultured with HSM to determine whether hepatocytes could be separated from the 201B7 cells. At 24 h after the 201B7 cells were cocultured with human primary hepatocytes in ReproFF (Figure 7A), the medium was changed to HSM. The 201B7 cells started to disappear at day 1 (Figure 7B), and cell number decreased at day 2 (Figure 7C). At day 3, all the 201B7 cells were abolished (Figure 7D).

## Discussion

If iPS cells could differentiate into hepatocytes, they would be useful for transplantation into patients with hepatic insufficiency. Elimination of iPS cells is necessary before the transplantation because iPS cell harbor tumorigenicity. In the present study, primary human hepatocytes were used because we did not have established a protocol of differentiation of iPS cells to hepatocytes. Our data clearly demonstrate that the HSM successfully separated primary human hepatocytes from coculture with iPS cells. The HSM is an ideal method to separate hepatocytes iPS cells when the protocol is established with regard to the differentiation of iPS cells to hepatocytes in the future.

Flow cytometry, which is commonly used to isolate target cells, was used by Yamamoto et al. to isolate hepatocytes differentiated from mouse embryonic stem (ES) cells [25]. They introduced ES cells with green fluorescent protein driven by an albumin promoter/enhancer. Albumin is expressed in endodermal cells as well [26]. The flow cytometry with albumin promoter/enhancer may isolate the other cells than hepatocytes, such as endodermal cells. Therefore, different strategy should be searched for. In addition, flow cytometry has been used to analyze surface antigens specific to hepatocytes. For example, delta-like 1 homolog (DLK1) has been used for the isolation of hepatoblasts [27]. The issue with DLK-1 is that the surface antigen is not expressed in the human adult liver [28]. Therefore, it may not be possible to isolate mature hepatocytes differentiated from hiPS cells with DLK-1. We have subsequently focused on methods to eliminate iPS or ES cells in our study. Sublethal heat shock induces apoptosis in human ES cells [29], but might damage differentiated cells intended for transplantation. Cheng et al. reported the same strategy with suicide genes [30]. They introduced a thymidine kinase gene driven by the Nanog promoter into hiPS cells. The cells were subsequently ablated with ganciclovir treatment. This method is ideal for differentiated hepatocytes because they do not express Nanog. One potential issue would be that ganciclovir might be toxic. Conesa et al. screened a library of 1120 small chemicals to identify molecules that cause mouse ES cells to undergo apoptosis [31], and found that benzethonium chloride and methylbenzethonium cause apoptosis in human iPS and mouse ES cells but not in human fibroblasts or mouse embryonic fibroblasts. Both reagents are quaternary ammonium salts and are used as antimicrobial agents. The reagents may damage hepatocytes because they are also used in cancer therapy. *N*-oleoyl serinol (S18), which is a ceramide analogue, eliminates residual pluripotent cells in embryoid bodies [32]. Interestingly, S18 promotes neural differentiation of embryoid body-derived cells. This strategy is promising because the reagent not only eradicates undifferentiated cells but also promotes their differentiation toward target cell types. Our HSM is suitable for the elimination of hiPS cells because it does not contain hazardous reagents or introduce genetic material. Our results show that HSM abolishes hiPS cells after 3 days in culture. Prior to performing the experiments, we anticipated a difference in the viability of hiPS cells cultured in media with or without KSR dialysis or the addition of IDA. Unexpectedly, a dialysis of KSR or the addition of IDA had no effect on hiPS cell survival. As expected, the primary human hepatocytes survived in the HSM as well as in the HCM, which is a recommended medium.

Our results clearly demonstrate that the hiPS cell death in the HSM was due to apoptosis. The pluripotent cells remained undifferentiated, as indicated by the positive immunostaining with Nanog, SSEA-1, and TRA-1-60. We speculate that the hiPS cells could not utilize galactose or ornithine for gluconeogenesis or the urea cycle, respectively. As a result, shortage of energy and arginine induced apoptosis in the hiPS cells. To our knowledge, this is the first report of hiPS cells undergoing apoptosis and being eliminated in media without glucose or arginine although the media were supplemented with galactose and ornithine. This hypothesis is supported by the finding that when somatic cells are reprogrammed, glycolysis is stimulated and energy metabolism shifts [33].
