# Correction: Survival of Primary Human Hepatocytes and Death of Induced Pluripotent Stem Cells in Media Lacking Glucose and Arginine

**DOI:** 10.1371/annotation/52de2a01-7d5e-44d5-b6ce-ed440cdb4ad6

**Published:** 2014-01-07

**Authors:** Minoru Tomizawa, Fuminobu Shinozaki, Takao Sugiyama, Shigenori Yamamoto, Makoto Sueishi, Takanobu Yoshida

There is an error in Figure 2, part A. In the Day 2 row, the second image to the right is incorrect. Please view the correct figure here: http://plosone.org/corrections/pone.0071897.g002.cn.tif

